# Evaluating Alternatives to Cold Stabilization in Wineries: The Use of Carboxymethyl Cellulose, Potassium Polyaspartate, Electrodialysis and Ion Exchange Resins

**DOI:** 10.3390/foods9091275

**Published:** 2020-09-11

**Authors:** Maria Pilar Martínez-Pérez, Ana Belén Bautista-Ortín, Valerie Durant, Encarna Gómez-Plaza

**Affiliations:** 1 Department of Food Science and Technology, Faculty of Veterinary Sciences, University of Murcia, Campus de Espinardo, 30100 Murcia, Spain; mpilar.martinez2@um.es (M.P.M.-P.); anabel@um.es (A.B.B.-O.); 2 Enology advisor for Esencia Wine Cellars, Carretera MU-15-A, Km. 11, 5, Jumilla, 30520 Murcia, Spain; valedurand@hotmail.com

**Keywords:** wine, tartaric stabilization, carboxymethyl cellulose, potassium polyaspartate, ion exchange resins, electrodialysis

## Abstract

The tartaric stabilization of wines before bottling to avoid the precipitation of tartaric acid salts is an important and common step during wine production. The presence of precipitated salt crystals in bottled wines is detrimental to their quality and can even be a legal issue in some countries. Different methodologies are used in wineries to substitute the classical low-temperature stabilization process, which is an effective but costly process. This study comprises two years of experiments with red wines at an industrial scale. In the first year of the experiment, two subtractive methods (ionic exchange resins and electrodialysis) were tested, whereas two additive methods (potassium polyaspartate and carboxymethyl cellulose, both of them containing gum Arabic) were tested the second year. The tartaric stability of the wines, together with the oenological, chromatic and sensory characteristics, were followed during one year in the bottle. The results indicate that carboxymethyl cellulose and potassium polyaspartate (both combined with gum Arabic) were best at maintaining the sensory and chromatic characteristics during storage, with potassium polyaspartate providing a good tartaric stability to the treated wine and this wine being, in general, preferred in a sensory analysis test.

## 1. Introduction

Wine is an acid solution that contains tartaric acid as the main acid. This acid is found in wine, forming salts with the most abundant cations, potassium and calcium, or is in its ionized forms. The tartaric salts present in the wine are mainly potassium bitartrate (KHT), neutral potassium tartrate (TK_2_) and neutral calcium tartrate (TCa), with a lower concentration of potassium and calcium double tartrate (T_2_K_2_Ca), and the mixed salt of calcium malotartrate (MTCa_2_) being present too. The ionized forms of the tartrate (HT^−^) and (T^−2^) ions are also found [1].

Tartaric instability is the precipitation of these tartaric salts and their consequent presence in the bottom of the bottle in the form of crystals. The musts and young wines contain high amounts of tartaric salts, often close to the point of supersaturation. The increase in the concentration of ethanol during vinification and the storage at low temperatures may lead to the precipitation of these salts, potassium bitartrate being one of the salts with less solubility in hydroalcoholic solutions and therefore the major cause of this crystal formation [1,2].

This problem has been aggravated in recent years because the increase in average temperatures during the maturation period causes the grapes to have lower total acidity at the time of harvest and higher levels of cations, especially potassium [3]. Acidity can be corrected in the winery, but the high concentration of potassium will facilitate the apparition of tartaric salt crystals.

The formation of these crystals is not a problem that endangers the consumer’s health but can be a commercial problem in terms of non-acceptance by the consumers themselves and a legal problem in some countries, as reported by Eder et al. [4]. Consumers may assume the presence of these crystals as a defect, since it modifies the organoleptic perception and alters the cleanliness of the wines, which often appear cloudy. In addition, they are often confused with microbiological problems, additives or glass remnants.

The process of tartaric stabilization, carried out before wine bottling, seeks to ensure a good physicochemical stability by avoiding the precipitation of the aforementioned salts and ensuring a satisfactory commercialization of the product.

Some of the techniques used for tartaric stabilization are considered “subtractive” and involve the reduction of the concentration of ions responsible for the tartaric precipitation in wines, mainly potassium, while others are considered “additive” techniques and use protective colloids or crystallization inhibitors that can be added to the wine.

Cold treatment (or low temperature treatment) is the most widespread method of potassium tartaric salts stabilization used by wineries nowadays [5]. This process involves lowering the wine’s temperature close to its freezing point and storing it in an isothermal tank, for a variable length of time of 7–12 days for white and rose wines, and for two to three weeks for red wines, to force the precipitation of tartaric salts prior to wine bottling. Although this method has been proven to be very effective, it may lead to a loss of wine color compounds and compounds responsible for the aroma and present some significant disadvantages for wineries, such as a long treatment time, the high economic cost of the technique and the environmental problems caused by the generated waste [5,6]. The constant movement necessary for heat exchange in the contact process also causes the oxygen content in the wine to increase, which can cause a partial oxidation of the wine [7]. Furthermore, this technique can induce the simultaneous precipitation of polysaccharides and polyphenols, which can, in turn, affect the wine quality [8].

These are the reasons why other products and methodologies have been introduced in wineries. Among the subtractive processes, we can find electrodialysis (ED) and the use of cation exchange resins (IER). Electrodialysis (ED) is a process of separation and/or concentration of ions, through selective membranes with the application of a continuous current [9,10,11,12]. ED eliminates the cations that cause the precipitation of tartaric salts [10,13]. The overall electrical energy consumption required for stabilization by electrodialysis might be up to eight times lower than that required for cold stabilization [14]. An ED device consists of a central cell containing the product to be electrodialyzed (e.g., wine), separated from the anode and cathode compartments in which water flows, by anion- and cation-selective membranes. When an electric field is applied, the ions in the wine migrate towards the appropriate electrode [14]. The net effect of electrodialysis is similar to the cold stabilization, with a decrease in KHT. However, ED removes Ca^2+^ and other metals and thus is potentially more effective at preventing TCa instability. The resulting wines are perceived as being “fresh” and not so “heavy” in the mouth [10].

In the case of ion exchange systems, the ion exchange resin is an insoluble gel matrix that contains the ions capable of being exchanged with some ions from the liquid around them. To stabilize the wine, the potassium ions are replaced with hydrogen ions and, in this way, the potassium bitartrate salts are replaced by tartaric acid [6]. Typically, the operation involves treating only a small portion of the wine and then mixing the wine treated by the resins with the rest of the untreated wine. The proportion of treated wine can vary significantly depending on the nature of the wine, the resin employed and the organoleptic characteristics. Several studies have indicated that this treatment does not affect the organoleptic quality of wine [15,16], while other studies showed that the cation exchange treatment induced a negative evaluation of the organoleptic characteristics [17]. Thus, there has been conflicting evidence with regards to the potential effects of ion exchange resin treatment on the sensory characteristics of the wines.

The additive processes make use of protective colloids or crystallization inhibitors that can be added to the wine. Currently, metatartaric acid, carboxymethylcellulose (CMC), mannoproteins, gum Arabic and potassium polyaspartate (KPA) are the only additives that are permitted and used for wine tartaric stabilization. Amongst them, carboxymethyl cellulose and potassium polyaspartate are the most used nowadays.

Carboxymethylcellulose (CMC) or cellulose gum is a polysaccharide obtained from cellulose from plant cells. It acts as a protective colloid, preventing the growth of KHT crystals. It is insoluble in ethanol and, therefore, poorly soluble in wine, making it difficult to dose the product. To improve these aspects, effective liquid products have been developed [18]. The CMC is a good alternative to other additive methods because it is an inexpensive product that is not sensitive to temperature, a great advantage over other common stabilizing agent as the metatartaric acid. The International Organization of Vine and Wine approved the use of carboxymethylcellulose in doses of up to 200 mg/L (Resolution OIV/OENO 586/2019). CMC can act by complexing the potassium, decreasing the number of free ions involved in crystal formation [19,20], as well as decreasing the crystal growth rate [19].

According to this OIV resolution, the use of CMC in wine is currently limited to white and sparkling wines, because its application in red wines is reported to be less efficient and might lead to color loss and haze formation [21,22]. However, given the large advantages that its use presents and to prevent its instability in the presence of polyphenols, the use of CMC in combination with gum Arabic (CMC+GA) has been proposed for the stabilization of red wines. In young red wine, gum Arabic (GA) is used as a protective colloid in order to prevent or limit the aggregation and precipitation of unstable colloids as the coloring matter, binding the polyphenols and preventing their interaction and aggregation with proteins and other substances [23]. For the time being, the use of CMC has not been yet recommended by the OIV for red wine stabilization, although it should not be forgotten that not all the countries with an important winemaking industry are under the OIV regulations, such as the United States.

Potassium polyaspartate (KPA) is a biopolymer obtained from a condensation process of L-aspartic acid. The OIV approved its use in 2016. KPA has a negative charge when it is at wine pH, allowing to bind to the potassium (K^+^) cation that has a positive charge, limiting the formation and precipitation of the salts. It has been stated that it presents high advantages, such as having a stabilization capacity similar to that of metatartaric acid, but persistent over time; no toxicity to humans; no impact on the color or pigmentation of the wine, since it does not interact with the tannins, polyphenols or anthocyanins in wine; and no effect on the organoleptic properties of wine [24]. Even though no problems associated with its use in red wines have been reported, its combination with gum Arabic is an interesting option for these wines, to increase colloidal stability. Concerns were raised on the fact that the mineral binding properties of the KPA could be responsible for a reduction in microelement bioavailability in humans. For this reason, and for the protection of consumers’ health, the binding properties of potassium polyaspartate versus three minerals (calcium, iron and magnesium) were assayed and the results obtained by this research showed that, when potassium polyaspartate is added to wine, the negative charges of the additive get saturated, as expected by the specific role of KPA in tartaric stabilization. In conclusion, the effect on mineral bioavailability must be considered negligible [25].

Given the different techniques and products that may compete with the cold treatment protocol, and in order to continue to explore the possibilities of the use of CMC for red wines, the objective of this study was to study, at the winery scale, the use of IER and ED during the first year of the experiment and CMC and KPA (both products added as commercial preparations containing gum Arabic) during the second year, to determine their effect in the tartaric stability as well as in the chemical and sensory characteristics of red wines, looking for alternatives to the application of the cold stabilization method, to minimize the cost of the stabilization process and the negative effects on the quality, while maintaining an adequate tartaric stability of the wines.

## 2. Materials and Methods

### 2.1. Wines

The study was carried out with Monastrell red wine made by Esencia Wines (Jumilla, Murcia, Spain), in two different years, 2016 and 2017.

### 2.2. Stabilization Methods

The red wine from the 2016 vintage (36,000 L) was stabilized with two different subtractive methods, ion exchange resin (10,000 L) and electrodialysis (10,000 L), and the resulting wines were compared with the effect of stabilizing the wine with one additive method, carboxymethylcellulose (5000 L), and with the most used method, cold stabilization (10,000 L).

The red wine from the 2017 vintage (16,000 L) was stabilized with two different additive products, potassium polyaspartate (KPA) and carboxymethylcellulose (2500 L for each treatment were used, both additives containing also gum Arabic, and the resulting wines were compared with the effect of stabilizing the wine with one subtractive method, ion exchange resin (10,000 L). This year, the winery, with a clear commitment to a more sustainable production, did not use the cold stabilization method for treating any of its wines.

A control that had not undergone any stabilization process was used for each wine each year (1000 L each year).

For the treatment with ion exchange resins, a semiautomatic cation exchange system ISR (AEB Iberica, Barcelona, Spain) was used. The operating cycle lasted 4 h and took place in three phases: conditioning, regeneration and washing. In this type of treatment, only part of the wine is treated to mix it with the original wine until it reaches stability. The final wine (original + treated wine) was considered stable when the conductivity drop was less than 5% in the Boulton test. Based on this, the mixing percentages of our wines were as follows:

- Red wine 2016: 80% control wine + 20% wine treated with resins.
- Red wine 2017: 70% control wine + 30% wine treated with resins.

For stabilization by electrodialysis, a STARS Stab 15/30 system was used (Oenodia, Pertuis, France). Before the stabilization process, the initial conductivity of the wine was calculated to configure the equipment. The equipment had a total surface of 3 m^2^ and a treatment capacity of 15 hL/h. It had a discontinuous operation regime, circulating the wine between the membrane reactor and a tank, until the wine reached the desired conductivity; then, a new cycle would begin.

For cold stabilization, long-term stabilization was performed. For this, the freezing temperature of the wine was previously calculated to decide on the cooling temperature, following the commonly used formula: Temperature = (−°alcoholic/2) + 1. The red wine in 2016 had an alcoholic grade of 13.6% vol. Therefore, its freezing temperature was −5.8 °C. The temperature of this wine was lowered to −5.5 °C, and it was kept in an isothermal tank for 15 days.

The stabilization treatments using carboxymethylcellulose were carried out by the addition of liquid CMC with gum Arabic (CMC+GA, Stab Mega, Enartis, Barcelona, Spain) at a dose of 150 mL/hL just before bottling. The potassium polyaspartate stabilization treatment was carried out by addition of a liquid KPA preparation that also contained gum Arabic (KPA+GA, Zenith color, Enartis, Barcelona, Spain), at a dose of 200 mL/hL, which was also done at the moment of bottling.

After treatments and for both years, the wines were bottled and closed with cork stoppers, and stored at 20 °C in the dark. Samples were taken at 0, 6 and 12 moths, analyzing three bottles of each wine each time.

### 2.3. Analytical Methods

After the treatments, the wines were bottled and the analyses carried out in triplicate at the time of bottling, at six months and at twelve months after bottling.

The red wines were analyzed for pH and titratable acidity using the analytical methods recommended by the OIV [26]. The concentration of tartaric acid was measured using a colorimetric kit (EnzytecTM Color, Boehringer Mannheim R-Biopharm, Germany). The calcium and potassium ions were measured by inductively coupled plasma mass spectrometry (ICP-MS). Wine samples were previously filtered before the analysis and diluted (1:10, v/v) using 2% v/v HNO_3_. An Agilent 7900 (Agilent, Tokyo, Japan) instrument was used according to the conditions described in [27]. A calibration curve up to100 mg/L was used for K and Ca.

UV/Vis spectrophotometric methods were performed using the UV/Vis spectrophotometer HELIOS α (Thermo Electron Corporation, MA, EEUU) on samples previously filtered with 0.45 μm nylon filters. Color intensity was determined according to Glories [28], using cuvettes with a 0.2 cm optical path length. The total polyphenol index was calculated by measuring the absorbance at 280 nm of the wine diluted 100 times with cuvettes with a 1 cm optical path length [1]. Total anthocyanins were measured at 520 nm after adding 20 mL of 0.1 N HCL to 0.5 mL of wine [29] and the total tannins were determined by the methyl cellulose method [30], and expressed as mg/L of catechin equivalents.

The concentration and composition of the tannins were also determined by the phloroglucinolysis method described by Kennedy and Jones and Pastor del Rio and Kennedy [31,32], using 5 mL of wine that was evaporated it in a centrivap concentrator (Labconco, Kansas City, MO, USA) and re-dissolved after that in 3 mL of water and then passed through a C18-SPE column (1 g; Waters), previously activated with 10 mL of methanol followed by 20 mL of water. The cartridge was then washed with 20 mL of water, and the compounds of interest were eluted with 10 mL of methanol, evaporated and then dissolved in 1 mL of methanol. The methanolic extract was left to react with the phloroglucinolysis reagent (1:1) (a solution of 0.2 M HCl in methanol, containing 100 g/L phloroglucinol and 20 g/L ascorbic acid) in a water bath for 20 min at 50 °C and then combined with 2 volumes of 200 mM aqueous sodium acetate to stop the reaction. HPLC analysis followed the conditions described by Busse-Valverde et al. [33]. Proanthocyanidin cleavage products were estimated using their response factors relative to (+)-catechin, which was used as the quantitative standard.

### 2.4. Measurement of the Tartaric Stability of Wines

Tartaric stability was determined using the so-called Boulton Test [34]. This is an analytical essay that consists of a rapid precipitation of the potassium bitartrate crystals, which are supersaturated in wine. The sample of the wine under analysis is cooled to −4 °C and a rapid precipitation of the crystals is caused by the addition of 10 g/L of potassium bitartrate, estimating the decrease in potassium with a conductimetric method. When there is no more precipitation, the conductivity value remains constant. The sample will have at this time the characteristics of a stable wine, and the value of the final conductivity will be what corresponds to this stabilized wine. The difference between the conductivity before the addition of the bitartrate and the final one provides a measure of the potential stability with respect to the bitartrate. It is normally considered that, if this difference is less than 5% of the initial value, the wine is stable. A cryothermic system was used (JP Selecta, Barcelona, Spain).

### 2.5. Sensory Analysis

The sensory analysis was carried out in two ways: by means of a descriptive test and a triangular discriminative test. The organoleptic study was carried out during the process at 0 and 12 months.

The descriptive sensory analysis was carried out with six panelists, all with experience and trained in this type of analysis and in the parameters evaluated. All the wines were served at room temperature and were evaluated in individual booths. Quantitative evaluation of the descriptors was carried out using an intensity scale of 1 to 10.

Triangular tasting tests were also carried out, at 0 and 12 months, to determine the existence of detectable differences between two different samples. This test was carried out by 20 panelists. For this analysis, three samples were presented to the panelist, two of which were identical. Samples were presented in random order in coded, clear 125 mL official glasses. Each taster selected the sample that he/she considered different (forced election method). All the different wine comparisons were evaluated. Although the aim of this technique is not the determination of preferences, the preferred sample in each series was also specified. The percentage of preferences was calculated with the correct answers, discarding the preferences of the incorrect tests.

### 2.6. Statistical Analysis

All the data was processed with Statgraphic Centurion XV software. ANOVA variance analysis was performed for the different parameters analyzed, and separation of means by the Tukey test at 5%. For the multivariate analysis, a cluster analysis and principal component analysis were conducted.

The results of the triangular tests were interpreted according to the tables of Carpenter et al. [35], which indicate the amount of correct answers necessary to obtain differences with different degrees of significance levels.

## 3. Results and Discussion

### 3.1. Tartaric Stability Test

Two subtractive methods (ED and IER) were applied in a 2016 red wine and compared with the most commonly used method, cold stabilization (CS), and also with an additive method, carboxymethyl cellulose + gum Arabic (CMC+GA), added to the wine prior to bottling.

**Figure 1:**
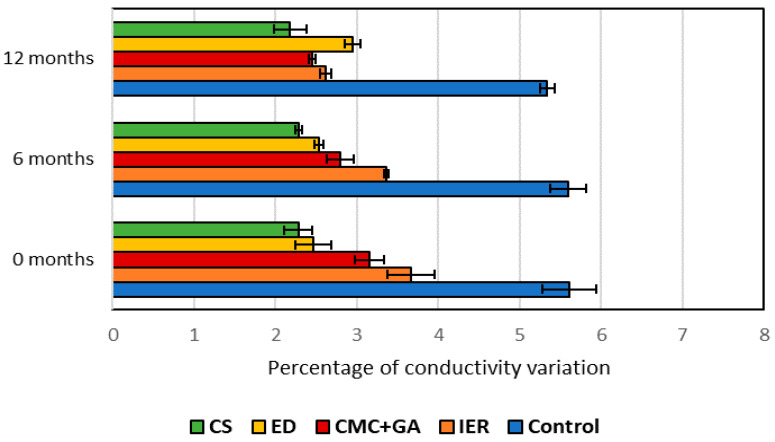
Tartaric stabilization test for the 2016 red wines. CS: cold stabilization; ED: electrodialysis; CMC+GA: carboxymethyl cellulose + gum Arabic; IER: ion exchange resins. The error bars represent the standard deviation.

**Figure 2:**
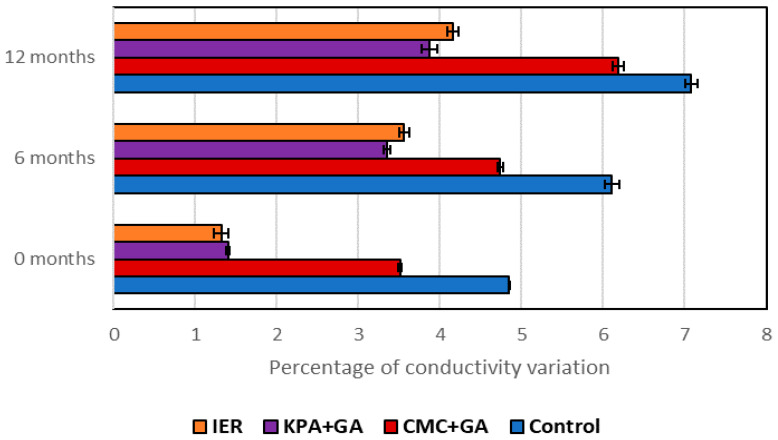
Tartaric stabilization test for the 2017 red wines. IER: ion exchange resins; KPA+GA: potassium polyaspartate + gum Arabic; CMC+GA: carboxymethyl cellulose + gum Arabic. The error bars represent the standard deviation.

Figure 1 and Figure 2 show the values obtained with the tartaric stability test just after the treatments (t = 0), at six months and at twelve months after bottling.

All treatments stabilized the wine, especially the cold treatment, and the wine maintained its stability after a year in the bottle. When looking at the two subtractive methods, the one that led to the most stable wine was ED, although after a year, the wine treated with IER showed the lowest values in the stability test (highest stability). Lasanta et al. [15] also found that IER-treated wines showed a high tartaric stability; however, they only measured the wines four weeks after treatment.

In 2017, the red wine stabilizations were carried out by two additive methods (carboxymethyl cellulose + gum Arabic and potassium polyaspartate + gum Arabic) and the results were compared with the wine treated with a subtractive method (ion exchange resins). After the treatment, the three methods stabilized the wine, the IER leading to the most stable wine. Looking at the two additive methods, the one that best stabilized the wine was potassium polyaspartate; it was also the only one that maintained the wine stable after a year of storage in the bottle. Bosso et al. [36] also found that KPA was a very good tartaric stabilizer for both red and white wines. Wines treated with carboxymethyl cellulose lost stability twelve months after treatment.

Several studies have shown that CMC, even without the aid of GA, can be a KHT crystallization inhibitor suitable for red wines [19,20,37,38]; however, the efficiency of this additive for tartaric stabilization depends on the chemical composition of the wine as well as doses [39], and our results showed how in 2016 the wines treated with CMC+GA remained stable after one year in the bottle but the same result was not observed in 2017, even though the tartaric acid, Ca and K concentration (Table 1 and Table 2) were lower in this wine. The inhibitory effect of KPA+GA remained after one year in the wine and similar results were observed by Canuti et al. [40] with KPA and Bosso et al. [36] working with KPA combined with GA.

### 3.2. Physico-Chemical Parameters

The physico-chemical results of the wines of 2016 and 2017 and their evolution over time are shown in Table 1 and Table 2.

Regarding the 2016 wines, it can be observed that, in general, the pH decreases after the subtractive treatments with ED and IER, remaining stable in the wines treated both with cold stabilization and with the CMC+GA treatments. The lower pH of the wines treated with the IER and ED methods can be an additional advantage for wines from warm areas, which usually present a high pH and low acidity. Total acidity increased with IER, due to the exchange that occurs between the wine and the resin, where the resin captures the K^+^ and Ca^2+^ cations and gives H^+^ protons to the wine, acidifying it. This effect has been observed by many other authors [6,15]. No changes were observed in total acidity with the ED, as with this method the cations that cause the tartaric precipitation are removed from the wine, using selective membranes but also the tartrate anion [41].

The conductivity value only decreased in wine treated with ED, correlating with a decrease in potassium concentration. The conductivity did not change in the IER-treated wine probably due to only a small portion of the wine being treated with the IER.

None of the treated wines differed in tartaric acid concentration from the control wine. With regard to Ca^+^^2^ and K^+^, all the three subtractive treatments led to wines with a lower concentration of these cations, IER and CS, reducing the K^+^ contents more prominently than ED, results also observed by Corti and Paladino [42].

It is important to note that when the wine was treated with CMC+GA, the physico-chemical composition of the treated wines barely differed from the control wine.

The results in Table 1 also show that the evolution of the composition of the wines was not affected by the treatments, with all wines evolving similarly. Over time, a decrease in pH, a slight increase in total acidity, a decrease in conductivity and a decrease in tartaric acid and Ca^+2^ and K^+^ was a commonly observed event. The decrease in tartaric acid and Ca^+^^2^ and K^+^ was not correlated with an instability of the wines, as observed in Figure 1, and it was difficult to understand. Thinking of the reasons behind these values, we hypothesized that the filtration step previous to the ICP-MS analysis of the Ca and K eliminated the small, suspended potassium bitartrate crystals and colloidal material (condensed phenolic compounds and other colloids) from the red wine, were some of the cations could be bound to.

For the 2017 experience, the additive methods, CMC+GA and KPA+GA, changed the wine composition less than the subtractive methods, since no changes in pH, total acidity and tartaric acid concentration could be observed, only a slight increase in conductivity in KPA+GA, maybe due to some liberation of K^+^ since the concentration of Ca^+^^2^ and K^+^ was slightly higher in the treated wines. This increase in Ca^+^^2^ and K^+^ was also observed in the CMC+GA-treated wines. Guise et al. [18] reported that some CMS’s may also contain important quantities of potassium in their composition. Comparing results, the IER methodology promoted larger changes in the treated wine, and, similar to the changes observed in 2016, a decrease in pH, increase in total acidity, decrease in conductivity and decrease in Ca^+^^2^ and K^+^ were observed.

### 3.3. Chromatic and Phenolic Parameters

The chromatic results of the wines and their evolution over time are found in Table 3 and Table 4.

Regarding the 2016 red wines, the two subtractive methods, ED and IER, decreased the wine color intensity although the cold stabilized wine and the CMC+GA-treated wine showed a more significant decrease. The total phenol index (TPI) also decreased when the wines were treated with ED and CS. Gómez-Benítez et al. [40] also reported a decrease in color intensity in cold-stabilized wines. The decrease in chromatic parameters in the wine treated by electrodialysis could be due to the adsorption of high molecular weight compounds in the membranes, as described by Bdiri et al. [43]. Some of these compounds, such as polysaccharide polymers, prevent tannin aggregation and precipitation and their elimination could facilitate a decrease in color intensity [44,45]. However, contrary to our findings, other authors have reported that electrodialysis does not affect wine color [42]. Although no differences were observed in TPI, IER was the technique that reduced the most the total anthocyanins content, a very important decrease being observed after the treatment, probably due to the retention of pigments on the surface of the resin, which is in accordance with the results obtained by other authors [16,17]. Ibeas et al. [6] also found that the use of IER for the stabilization of red wines caused a significant decrease, not only of anthocyanins, but also of the different proanthocyanidin fractions, coincident with our results since a decrease in tannins was also observed.

If we compare these techniques with an additive technique, CMC+GA, we could find that it also caused chromatic changes in the wine, causing the color intensity to decrease, even though the total polyphenols, total anthocyanins and total tannins remained quite stable. The use of GA did not avoid this decrease in color intensity. Several previous studies have reported that the use of CMC in red wines may decrease their color intensity [19,20], an observation coincident with our results, even though CMC was combined with gum Arabic for avoiding this decrease. Claus et al. [20] tested the use of CMC in different red wines and some wine samples, especially those with a higher color intensity and protein content, developed a turbidity and a precipitate, composed of pathogenesis-related wine proteins and colored matter, was formed.

With respect to total tannins, the treatment that most negatively affected its concentration was ED followed by IER and CS. Lasanta et al. [15] also reported a slight decrease in the total tannin content of wines stabilized by cation exchange resin and Bdiri et al. [43] an adsorption of high molecular weight compounds in the ED membranes.

After 12 months of storage, CS wines presented the lowest color intensity, total phenol content and tannin content. Although the wine treated with electrodialysis was the one that best retained the original chromatic characteristics of the wine at the time of treatment, its evolution over time was the least favorable. The decrease in total anthocyanins at twelve months of treatment was the most significant of all treatments, it being almost 57%, very similar to the decrease in the total anthocyanins of the control wine (55%), compared to a 16% decrease in the IER-treated wine. This wine, although suffering a large loss of anthocyanins after the treatment, showed a very stable behavior during storage.

Regarding the 2017 red wines, the two additive techniques caused similar changes in the chromatic composition of the wines, compared to a control wine without a stabilization treatment.

The two additive techniques cause a decrease in color intensity, especially in CMC-treated wines, even though both products were combined with GA. On the other hand, no clear changes in total polyphenols were observed, and there were only small changes in the total anthocyanins and total tannins, contrary to the results of Pittari et al. [37], who reported that wines treated with CMC showed significantly higher values of color intensity compared to the control wine and that the precipitation of colored pigments due to the addition of CMC was not a trend for all red wines. These different trends could be due to the fact that differences in wine protein content may play an important role in the extent of color loss in CMC-treated wines, as observed by Sommer et al. [46]. These techniques have also been compared with a subtractive technique, IER, which caused the same changes in the wine as the two additive techniques; that is, a decrease in color intensity, although much more significant than that observed with KPA+GA. The decrease in total anthocyanins observed in 2016 in the IER-treated wine was not observed in the 2017 treated wine.

Twelve months after the treatments, the wines treated with KPA+GA and CMC+GA maintained their color intensity, with a small decrease in total anthocyanins and total tannins. The presence of GA in the commercial preparations may help to improve the stability of the wine color compounds over time. The IER-treated wine also maintained its chromatic characteristics similar to those of the control untreated wine.

In order to have a deeper knowledge of the composition of the tannins after twelve months in the bottle and how the different stabilization treatments affected this composition, a tannin analysis, using the phloroglucinolysis reagent, was conducted in the wines after 12 months in the bottle (Table 5 and Table 6).

This analysis gives us information not only of the tannin concentration but also the mean degree of polymerization. However, the values of the tannin concentration obtained with this method are lower than with the methylcellulose method since the chemical changes occurring during wine ageing leads to the formation of new covalent bonds between the units and some of them are resistant to acid-catalyzed cleavage and, therefore, not determined with this methodology [47]. In 2016, the highest concentration of easily depolymerizable tannins was found in the control wine and the wine treated by CMC+GA, the CS treatment leading to a wine with the lowest concentration (as also observed when tannins were measured with the methyl cellulose method) and with the lowest mean degree of polymerization (mDP), indicating a possible precipitation of the polymers with the highest molecular weight.

In the 2017 wines and after 12 months in the bottle, the observed variations in tannin content between the different wines were lower, the KPA+GA-treated wine being the only wine that significantly differed from the results of the control wine, with a lower concentration of tannin, as also observed in Table 4, although in this case, the values were not significantly different from the control wine. Contrary to these results, Bosso et al. [36] reported an increase in the flavonoid compounds after 12 months, when KPA+GA was used to treat the red wine. The mean degree of polymerization was quite similar for all the wines, only slightly higher in the CMC+GA-treated wine.

### 3.4. Multivariate Analysis

In order to try to group all the information given by the chemical and chromatic parameters, and, in this way, acquire information on how different treated wines were from the control wine, a multivariate analysis (principal component analysis) was conducted. The principal component analysis (PCA analysis) is mostly used as a tool in exploratory data analysis and it is often used to visualize distance and relatedness between populations and how much the different variables weigh in these groupings.

**Figure 3:**
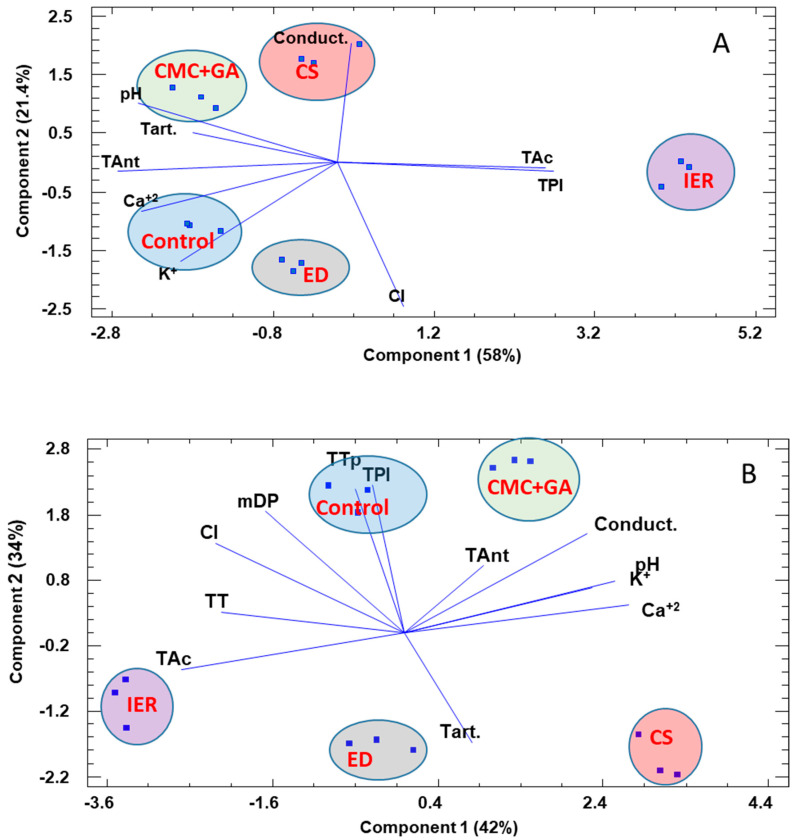
Results of the PCA analysis for the 2016 red wines at the time of bottling (**A**) and after 12 months in the bottle (**B**). ED: electrodialysis; IER: ion exchange resins; CS: cold stabilization; CMC+GA: carboxymethyl cellulose + gum Arabic; CI: color intensity; TPI: total phenol index; TAnt: total anthocyanins; Tac: total acidity; Tart: tartaric acid; TT: total tannins; TTp: total tannins by phloroglucinolysis; Tac: total acidity; Conduct: conductivity. The percentage of variance explained by each component is given in parenthesis.

**Figure 4:**
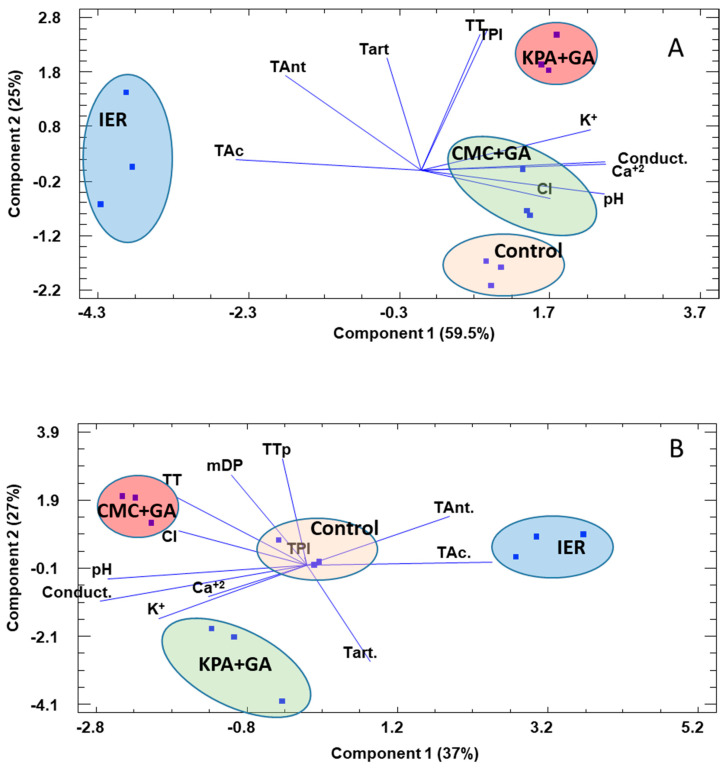
Results of the PCA analysis for the 2017 red wines at the time of bottling (**A**) and after 12 months in the bottle (**B**). CMC+GA: carboxymethyl cellulose + gum Arabic; KPA+GA: potassium polyaspartate + gum Arabic; IER: ion exchange resins.; CI: color intensity; TPI: total phenol index; TAnt: total anthocyanins; TT: total tannins; TAc: total acidity; Conduct: conductivity; Tart: tartaric acid. The percentage of variance explained by each component is given in parenthesis.

Figure 3 and Figure 4 show the results of this multivariate analysis at time 0 and after 12 months in the bottle. Both at t = 0 and t = 12, it could be observed how the IER-treated wine was clearly separated from the other wines along the *x*-axis (PC1), especially due to its high acidity and low pH, low anthocyanin content and low quantities of Ca^+2^ and K+. This difference was very important at t = 0. The other wines were separated along the *y*-axis (PC2), with the ED-treated wine being closer to control wine, in the negative part of PC2, whereas the CMC+GA- and CS-treated wines were located in the positive part of PC2. After 12 months, the CMC+GA-treated wine was now closely located to the control wine; this wine maintaining a high total tannin content (determined by phloroglucinol), total phenol index, total anthocyanins and color intensity. The IER wines were again clearly separated based in their high acidity, low pH, conductivity, Ca^+2^ and K^+^.

In the 2017 wines, the control wine was closely located to the CMC+GA-treated wine, and again, as in 2016, the IER-treated wine was the most different wine. The separation of the IER samples was mainly based on the variables of total acidity, due its high values, and the low values of pH, conductivity, Ca^+2^ and K^+^. The KPA+GA-treated wine was located in the positive part of PC2, due to a higher total phenol index along with a higher K^+^, conductivity and color intensity. After 12 months, the clear separation of the wine samples treated with IER was very evident and the KPA+GA- and CMC+GA-treated wines were located in the negative part of PC 1, due to their higher chromatic data.

### 3.5. Sensory Analysis

**Figure 5:**
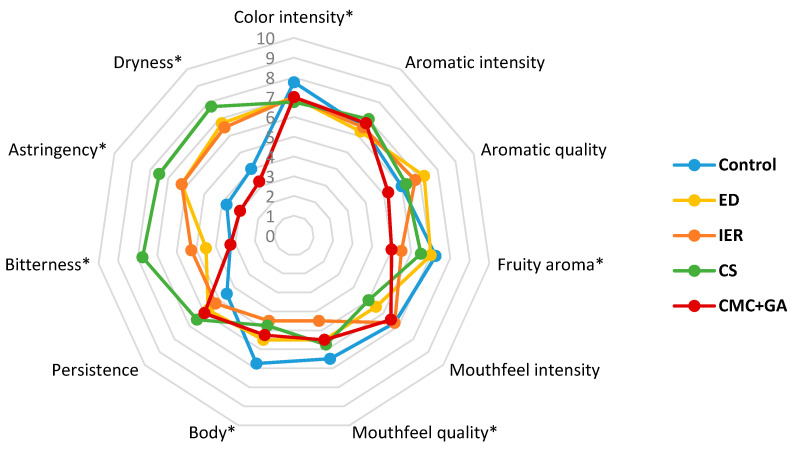
Results of the descriptive test in the 2016 red wine at the time of bottling (time 0). ED: electrodialysis; IER: ion exchange resins; CS: cold stabilization; CMC+GA: carboxymethyl cellulose + gum Arabic. * Statistically significant differences with respect to the control.

**Figure 6:**
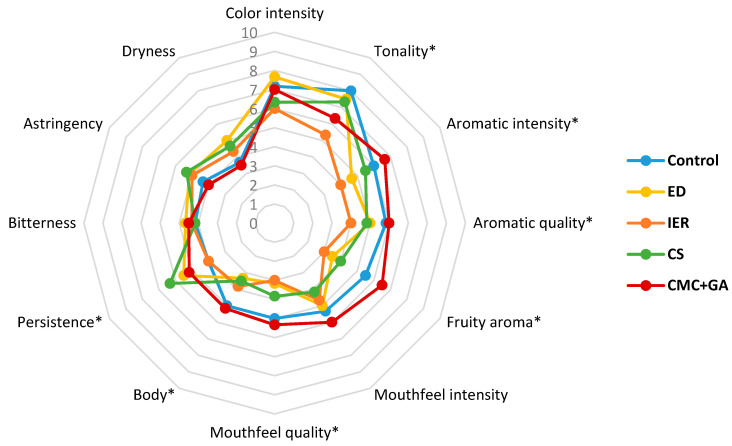
Results of the descriptive test in the 2016 red wine at twelve months in the bottle. ED: electrodialysis; IER: ion exchange resins; CS: cold stabilization; CMC+GA: carboxymethyl cellulose + gum Arabic. * Statistically significant differences with respect to the control.

**Figure 7:**
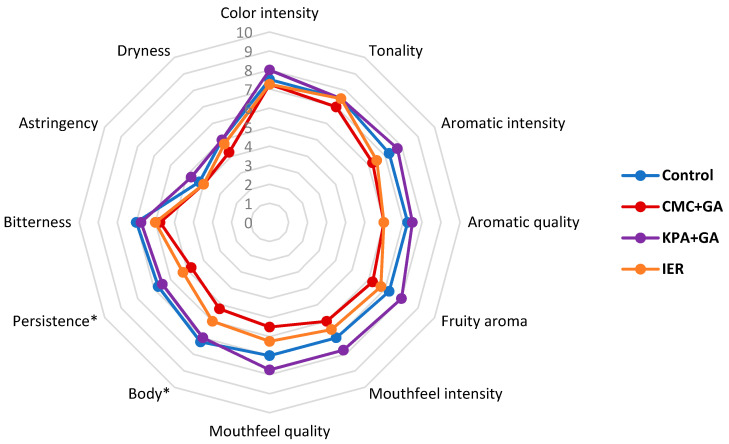
Results of the descriptive test in the 2017 red wine at the time of bottling (time 0). CMC+GA: carboxymethyl cellulose + gum Arabic; KPA+GA: potassium polyaspartate + gum Arabic; IER: ion exchange resins. * Statistically significant differences with respect to the control.

**Figure 8:**
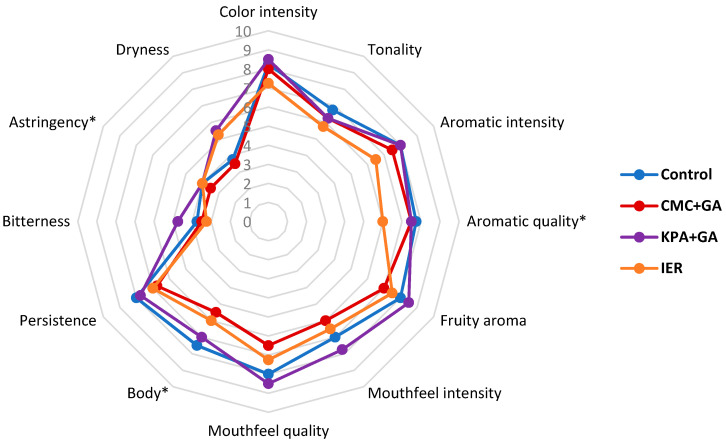
Results of the descriptive test in the 2017 red wine at twelve months in the bottle. CMC+GA: carboxymethyl cellulose + gum Arabic; KPA+GA: potassium polyaspartate + gum Arabic; IER: ion exchange resins. * Statistically significant differences with respect to the control.

Table 7 and Table 8 show the results of the triangular test carried out with 20 panelists in the 2016 and 2017 wines, at zero and twelve months, whereas Figure 5, Figure 6, Figure 7 and Figure 8 show the results of the descriptive sensory analysis, carried out with six trained panelists.

In 2016 and at t = 0, only three comparisons, namely, the control wine vs. IER-treated wine, CMC+GA-treated wine vs. IER-treated wine and CMC+GA-treated wine vs. CS-treated wine, were significantly different and the IER-treated wine was never preferred. Similar to our findings, Corti and Paladino [42] found no significant sensory preferences when ED- and CS-treated wines were compared with a control wine.

After 12 months in the bottle, the panelists could better distinguish the wines. Now, only the control vs. CMC+GA-treated wines and CMC+GA- vs. ED-treated wines were not significantly distinguished, which is quite in agreement with the results of the multivariate analyses. The panelists always preferred the control wine, and the CMC+GA-treated wine versus the IER- and CS-treated wines. Comparing the subtractive methods, the IER wine was never preferred. Contrary to that, other authors have reported that they did not find significant sensory differences between wines treated with exchange resin and the control wines and that this operation did not reduce the organoleptic quality [16].

Figure 5 and Figure 6 are coincident with the triangular test and showed that differences with the control wine were more evident with the subtractive methods than with the CMC+GA-treated wine. The CMC+GA-treated wine only differed from the control wine in fruity aroma whereas much higher values of dryness, bitterness and astringency were scored in the wines treated with the subtractive methods, which significantly differed from the control wine. After 12 months, differences were mainly observed in aroma and mouthfeel quality, the subtractive treated wines being scored lower. At this point, differences in mouthfeel attributes diminished.

Regarding the 2017 wines, at t = 0, only the control vs. KPA+GA-treated wines and CMC+GA- vs. KPA+GA-treated wines were not distinguished. The control was preferred over the CMC+GA and IER-treated wines, and the CMC+GA- and KPA+GA-treated wines were much preferred over IER. After 12 months in the bottle, the results were the same.

Figure 7 and Figure 8 show the results of the descriptive analysis for the 2017 wines, at t = 0 and t = 12 months.

The results showed how the use of additive methods led to smaller differences in the sensory attributes of the wine than that observed with the subtractive methods, both at t = 0 and t = 12 months, with the KPA+GA-treated wine showing better sensory scores than the control wine in most of the aroma and mouthfeel attributes. The presence of GA could contribute to the positive mouthfeel characteristics of this wine although the same results were not observed in the CMC+GA-treated wines, with lower scores than the control and KPA+GA wines in most of the attributes.

## 4. Conclusions

Four different stabilization methods were tested in two red wines in a large-scale winery, to determine which method could maintain a high wine tartaric stability while modifying the least the physico-chemical, chromatic and sensorial characteristics of the wines, and, therefore, which one could be proposed as the best substitute for the cold stabilization method, the most common method practiced by many winemakers but a time consuming and costly process.

All the results pointed to the additive methods (CMC and KPA, both of them formulated with the addition of GA to better maintain the red wine’s chromatic characteristics), being those that modify the wine characteristics the least, whereas the IER-treated wines, although giving very good results in the tartaric stability tests, were those that showed the most important changes in the wine composition, being also the wines with the lowest scores in the sensory analysis and the least preferred wines.

The CMC+GA- and KPA+GA-treated wines presented very similar characteristics to the control wines. However, the use of CMC, only recommended at this moment by the OIV for white and sparkling wines, led to a certain decrease in wine color intensity just after the treatment and, moreover, in 2017 it did not maintain the wine tartaric stability after 12 months in the bottle, whereas the results of the evolution of the wine stability and physico-chemical, chromatic and sensory characteristics point to the use of KPA+GA as the best alternative to the cold stabilization process in wineries for red wine tartaric stabilization.

Most wineries are committed to the use of ecofriendly, energetically efficient processes, this while maintaining the wine quality and stability. Therefore, the substitution of the cold stabilization process is a main objective for them. This study presents useful practical information for the enological sector since it has been conducted in an industrial winery, dealing with volumes and conditions usually found in commercial wineries and, in addition, the characteristics of the treated wines were followed for one year. Similar studies are scarce, especially those involving the use of KPA, since it was approved quite recently, so information on its use and its long-term behavior can be of great interest.

## Figures and Tables

**Table 1:** Physico-chemical parameters measured in the 2016 red wines.

Storage Time	Wine	pH	Total Acidity (g/L)	Conductivity (mS/cm)	Tartaric Acid (g/L)	Ca (ppm)	K (ppm)
**0 Months**	**Control**	3.72 c	5.13 a	2.41 b	6.17 ab	193.36 d	1680.16 c
	**ED**	3.64 b	5.21 a	2.17 a	5.60 a	178.48 c	1518.22 b
	**IER**	3.52 a	5.94 b	2.41 b	5.29 a	148.26 a	1244.55 a
	**CS**	3.74 c	5.16 a	2.43 b	5.47 a	159.16 b	1220.98 a
	**CMC+GA**	3.72 c	5.25 a	2.45 b	7.00 b	190.38 d	1498.74 b
**6 Months**	**Control**	3.57 c	5.45 bc	2.35 b	4.63 b	106.78 b	1345.59 c
	**ED**	3.48 b	5.37 b	2.15 a	4.26 a	88.13 a	1090.39 a
	**IER**	3.35 a	5.71 d	2.13 a	5.93 d	86.29 a	1076.61 a
	**CS**	3.67 e	5.11 a	2.37 bc	4.35 a	118.00 c	1261.07 b
	**CMC+GA**	3.63 d	5.47 c	2.44 c	5.41 c	110.85 bc	1365.14 c
**12 Months**	**Control**	3.45 d	5.42 b	2.21 c	3.51 a	55.81 b	1081.83 b
	**ED**	3.37 b	5.68 c	2.00 b	3.76 ab	57.52 b	1118.98 c
	**IER**	3.24 a	6.17 d	1.98 a	4.04 bc	50.74 a	872.91 a
	**CS**	3.46 e	5.25 a	2.26 d	4.29 c	69.63 c	1085.15 b
	**CMC+GA**	3.42 c	5.48 b	2.39 e	3.73 ab	70.73 c	1131.00 c

ED: electrodialysis; IER: ion exchange resins; CS: cold stabilization; CMC+GA: carboxymethyl cellulose + gum Arabic. Different letters within the same column and same storage time indicate significant differences (*p* < 0.05).

**Table 2:** Physico-chemical parameters measured in the 2017 red wines.

Storage Time	Wine	pH	Total Acidity (g/L)	Conductivity (mS/cm)	Tartaric Acid (g/L)	Ca (ppm)	K (ppm)
**0 Months**	**Control**	3.84 b	5.31 a	2.44 b	4.78 a	76.98 b	1296.03 b
	**CMC+GA**	3.88 b	5.21 a	2.43 b	5.07 a	83.84 c	1558.54 c
	**KPA+GA**	3.82 b	5.27 a	2.51 c	5.46 a	82.58 c	1541.54 c
	**IER**	3.43 a	6.04 b	2.07 a	5.41 a	58.53 a	1061.86 a
**6 Months**	**Control**	3.79 b	5.08 a	2.34 b	4.15 ab	64.85 b	1247.75 b
	**CMC+GA**	3.80 b	5.30 a	2.49 c	4.75 a	70.75 c	1300.23 b
	**KPA+GA**	3.81 b	5.31 a	2.53 d	3.77 a	67.36 b	1313.15 b
	**IER**	3.5 a	6.14 b	1.92 a	4.17 ab	49.37 a	928.94 a
**12 Months**	**Control**	3.70 c	5.35 a	2.34 b	3.39 ab	49.39 a	963.31 a
	**CMC+GA**	3.70 c	5.45 a	2.60 c	3.27 a	55.71 bc	1053.09 b
	**KPA+GA**	3.65 b	5.62 b	2.64 c	3.80 b	56.76 c	1089.06 c
	**IER**	3.29 a	6.26 c	1.83 a	3.56 ab	52.23 ab	969.83 a

IER: ion exchange resins; KPA+GA: potassium polyaspartate + gum Arabic; CMC+GA: carboxymethyl cellulose + gum Arabic. Different letters within the same column and same storage time indicate significant differences (*p* < 0.05).

**Table 3:** Chromatic and phenolic parameters measured in the 2016 red wines.

RED WINES 2016		CI	TPI	TA (mg/L)	TT (mg/L)
**0 Months**	**Control**	13.33 d	65.93 b	429.03 b	2134.46 c
	**ED**	12.48 c	62.63 a	424.85 b	1934.39 a
	**IER**	12.75 c	65.52 b	251.88 a	2089.56 b
	**CS**	9.60 b	62.53 a	399.44 b	2024.63 b
	**CMC+GA**	9.58 a	64.47 b	424.85 b	2205.56 c
**6 Months**	**Control**	12.78 e	61.87 b	267.27 b	1905.36 c
	**ED**	11.22 c	62.58 c	260.12 b	1899.39 b
	**IER**	12.48 d	62.01 b	238.03 a	2100.92 d
	**CS**	8.53 a	58.40 a	266.25 b	1616.12 a
	**CMC+GA**	10.15 b	62.66 c	289.82 c	1882.21 b
**12 Months**	**Control**	12.60 d	62.87 c	193.31 a	1890.01 ab
	**ED**	10.50 b	58.37 a	182.48 a	1875.70 ab
	**IER**	11.80 c	60.73 b	211.70 b	2031.42 b
	**CS**	8.36 a	57.78 a	215.45 b	1581.32 a
	**CMC+GA**	10.88 b	65.17 d	244.15 c	1798.56 ab

ED: electrodialysis; IER: ion exchange resins; CS: cold stabilization; CMC+GA: carboxymethyl cellulose + gum Arabic; CI: Color Intensity; TPI: total phenol index; TA: total anthocyanins; TT: total tannins. Different letters within the same column and same storage time indicate significant differences (*p* < 0.05).

**Table 4:** Chromatic parameters measured in the 2017 red wines.

2017 RED WINES		CI	TPI	TA (mg/L)	TT (mg/L)
**0 Months**	**Control**	14.12 c	71.02 a	326.06 a	1973.05 a
	**CMC+GA**	12.29 a	73.02 a	328.64 a	2059.31 ab
	**KPA+GA**	13.50 b	75.34 a	362.81 b	2450.10 b
	**IER**	12.71 a	72.32 a	386.65 b	2062.49 ab
**6 Months**	**Control**	12.89 a	70.88 b	301.76 b	1920.16 a
	**CMC+GA**	13.15 b	74.70 c	300.51 b	2034.53 a
	**KPA+GA**	13.16 b	67.67 a	252.26 a	1957.25 a
	**IER**	12.78 a	70.34 b	303.57 b	2036.23 a
**12 Months**	**Control**	12.98 a	70.66 a	238.18 a	1701.91 a
	**CMC+GA**	13.85 c	70.20 a	251.65 a	1962.13 b
	**KPA+GA**	13.37 b	70.86 a	241.60 a	1638.83 ab
	**IER**	13.24 b	70.56 a	268.34 a	1609.55 a

CMC+GA: carboxymethyl cellulose + gum Arabic; KPA+GA: potassium polyaspartate + gum Arabic; IER: ion exchange resins; CI: color intensity; TPI: total phenol index; AT: total anthocyanins; TT: total tannins. Different letters within the same column and same storage time indicate significant differences (*p* < 0.05).

**Table 5:** Wine tannin concentration and composition, as measured by high performance liquid chromatography by the phloroglucinolysis method in the 2016 red wines after twelve months in the bottle.

RED WINES 2016	TTp (mg/L)	mDP
**Control**	768.15 c	5.16 c
**ED**	558.58 b	3.84 b
**IER**	613.68 b	5.09 c
**CS**	524.77 a	3.61 a
**CMC+GA**	719.58 c	5.13 c

ED: electrodialysis; IER: ion exchange resins; CS: cold stabilization; CMC+GA: carboxymethyl cellulose + gum Arabic. TTp: total tannins; mDP: medium degree of polymerization. Different letters within the same column indicate significant differences (*p* < 0.05).

**Table 6:** Tannins measured by high performance liquid chromatography by the phloroglucinolysis method in the 2017 red wines at twelve months in the bottle.

2017 RED WINES	TTp (mg/L)	mDP
Control	1037.04 b	3.74 ab
CMC+GA	1091.49 b	3.85 b
KPA+GA	875.7 a	3.66 a
IER	1013.09 b	3.73 ab

CMC+GA: carboxymethyl cellulose + gum Arabic; KPA+GA: potassium polyaspartate + gum Arabic; IER: ion exchange resins; TTp: total tannins; mDP: medium degree of polymerization. Different letters within the same column indicate significant differences (*p* < 0.05).

**Table 7:** Results of the triangular test and preference test carried out with 20 panelists in the 2016 red wine at the time of bottling (time 0) and after 12 months.

	0 Months		12 Months	
	Number of Right Answers	Preferences	Number of Right Answers	Preferences
Control vs. CMC+GA	10		10	
Control vs. IER	14 ***	Control (8)	14 ***	Control (10)
Control vs. ED	8		12 *	Control (8)
Control vs. CS	6		16 ***	Control (10)
CMC+GA vs. IER	11 *	CMC+GA (6)	13 **	CMC+GA (7)
CMC+GA vs. ED	7		10	
CMC+GA vs. CS	11 *	CMC+GA (9)	11 *	CMC+GA (9)
IER vs. ED	7		11 *	ED (6)
IER vs. CS	9		17 ***	CS (14)
ED vs. CS	10		13 **	CS (8)

ED: electrodialysis; IER: ion exchange resins; CS: cold stabilization; CMC+GA: carboxymethyl cellulose + gum Arabic. * *p* < 0.05, ** *p* < 0.01, *** *p* < 0.001.

**Table 8:** Results of the triangular discriminative and preference test carried out with 20 tasters for the 2017 red wine at the time of bottling (time 0) and after 12 months.

	0 Months		12 Months	
	Number of Right Answers	Preferences	Number of Right Answers	Preferences
Control vs. CMC+GA	11 *	Control (7)	12 *	Control (6)–CMC (6)
Control vs. KPA+GA	6		8	
Control vs. IER	19 ***	Control (18)	16 ***	Control (15)
CMC+GA vs. KPA+GA	10		8	
CMC+GA vs. IER	18 ***	CMC (16)	11 *	CMC (9)
KPA+GA vs. IER	17 ***	KPA+GA (17)	17 ***	KPA (16)

CMC+GA: carboxymethyl cellulose + gum Arabic; KPA+GA: potassium polyaspartate + gum Arabic; IER: ion exchange resins. * *p* < 0.05, *** *p* < 0.001.
